# A prospective, longitudinal, case–control study to evaluate the neurodevelopment of children from birth to adolescence exposed to COVID-19 in utero

**DOI:** 10.1186/s12887-023-03858-w

**Published:** 2023-01-30

**Authors:** Rachel A. Hill, Atul Malhotra, Vathana Sackett, Katrina Williams, Michael Fahey, Kirsten R. Palmer, Rod W. Hunt, Hayley Darke, Izaak Lim, Vesna Newman-Morris, Jeanie L. Y. Cheong, Clare Whitehead, Joanne Said, Paulo Bignardi, Evelin Muraguchi, Luiz Carlos C. Fernandes, Carlos Oliveira, Suresh Sundram

**Affiliations:** 1 Department of Psychiatry, School of Clinical Sciences at Monash Health, Monash Medical Centre, Monash University, Level 3, 27-31 Wright St, Clayton, VIC 3168 Australia; 2 Florey Institute for Neuroscience and Mental Health, University of Melbourne, Parkville, VIC Australia; 3 Department of Paediatrics, Monash University, Clayton, VIC Australia; 4 Department of Paediatrics, The University of Melbourne, Parkville, VIC Australia; 5 Department of Obstetrics and Gynaecology, The University of Melbourne, Parkville, VIC Australia; 6 Monash Women’s, Monash Health, Clayton, VIC Australia; 7 Department of Obstetrics and Gynaecology, Monash University, Clayton, VIC Australia; 8 Clinical Sciences, Murdoch Children’s Research Institute, Parkville, VIC Australia; 9 Monash Medical Centre, Early in Life Mental Health Service, Monash Health, Clayton, VIC Australia; 10 Department of Neonatal Services, Royal Women’s Hospital, Parkville, VIC Australia; 11 Department of Obstetrics and Gynaecology, Royal Women’s Hospital, Parkville, VIC Australia; 12 Maternal Fetal Medicine, Joan Kirner Women’s & Children’s at Sunshine Hospital, Western Health, Sunshine, VIC Australia; 13 School of Medicine, Pontifical Catholic University of Paraná, Londrina, Paraná, Brazil; 14 Mental Health Program, Monash Health, Melbourne, VIC Australia

**Keywords:** COVID-19, SARS-CoV2, Pregnancy, Neurodevelopment, Prospective

## Abstract

**Background:**

The Coronavirus disease (COVID-19) pandemic has created unprecedented acute global health challenges. However, it also presents a set of unquantified and poorly understood risks in the medium to long term, specifically, risks to children whose mothers were infected with the severe acute respiratory syndrome coronavirus 2 (SARS-CoV-2) during pregnancy. Infections during pregnancy can increase the risk of atypical neurodevelopment in the offspring, but the long-term neurodevelopmental impact of in utero COVID-19 exposure is unknown. Prospective, longitudinal studies are needed to evaluate children exposed in utero to SARS-CoV2 to define this risk.

**Methods:**

We have designed a prospective, case-controlled study to investigate the long-term impacts of SARS-CoV2 exposure on children exposed in utero. Women infected with SARS-CoV-2 during pregnancy will be recruited from Monash Health, the Royal Women’s Hospital and Western Health (Melbourne, Australia) and Londrina Municipal Maternity Hospital Lucilla Ballalai and PUCPR Medical Clinical (Londrina, Brazil). A control group in a 2:1 ratio (2 non-exposed: 1 exposed mother infant dyad) comprising women who gave birth in the same month of delivery, are of similar age but did not contract SARS-CoV-2 during their pregnancy will also be recruited. We aim to recruit 170 exposed and 340 non-exposed mother-infant dyads. Clinical and socio-demographic data will be collected directly from the mother and medical records. Biospecimens and clinical and epidemiological data will be collected from the mothers and offspring at multiple time points from birth through to 15 years of age using standardised sample collection, and neurological and behavioural measures.

**Discussion:**

The mapped neurodevelopmental trajectories and comparisons between SARS-CoV-2 exposed and control children will indicate the potential for an increase in atypical neurodevelopment. This has significant implications for strategic planning in the mental health and paediatrics sectors and long-term monitoring of children globally.

## Background

Historically, it is well documented that infections during pregnancy increase the risk for atypical neurodevelopment in offspring such as intellectual disability, cerebral palsy, autism and schizophrenia [1]. This has been noted in large epidemiological studies following influenza and measles epidemics, with varying degrees of severity depending on the pathogen and the gestation at the time of exposure to the infection [2]. A plausible but unknown prospect are severe long-term neurodevelopmental impacts following in utero exposure to SARS-CoV-2. This highly concerning prospect must be tested to establish the absolute risk and enable early intervention.

Transplacental or vertical transmission of SARS-CoV-2 has been reported [3]. Several case reports have confirmed the presence of SARS-CoV-2 in the amniotic fluid and umbilical cord blood [3–5], although this appears to be rare. Limited case studies also report elevated anti-SARS-CoV-2 Immunoglobulin M (IgM) and IgG antibodies and positive nasopharyngeal swab tests in neonates born to SARS-CoV-2 infected mothers [4–11]. However, while vertical transmission is rare, of considerable concern is the maternal immune response to SARS-CoV-2 and the so called ‘cytokine storm’ that is a common occurrence following infection. Concern over this immune response is borne from previous ecological studies, birth cohort studies and animal models that have established key links between the activation of pro-inflammatory pathways in the mother with adverse neurodevelopment outcomes in the infant [2, 12–18].

To this end, we have established a large-scale, multi-site international initiative to monitor the long-term neurodevelopmental outcomes of infants exposed to SARS-CoV-2 in utero. The aim of the study is to assess the neurodevelopmental outcomes for children exposed to SARS-CoV-2 in utero. We hypothesise that children of mothers who contracted SARS-CoV-2 infection during pregnancy will show a heightened risk for future neurodevelopmental disorders.

We describe here a prospective longitudinal protocol to assess children exposed to SARS-CoV-2 in utero at multiple key neurodevelopmental time points from birth to 15 years of age. This protocol was established at Monash University, Melbourne, Australia and adapted at the School of Medicine, Pontificia Universidade Catolica do Parana, Londrina, Brazil. We encourage international uptake of this protocol for standardised global monitoring of neurodevelopmental outcomes.

## Methods / design

### Aim and study setting

**Fig. 1:**
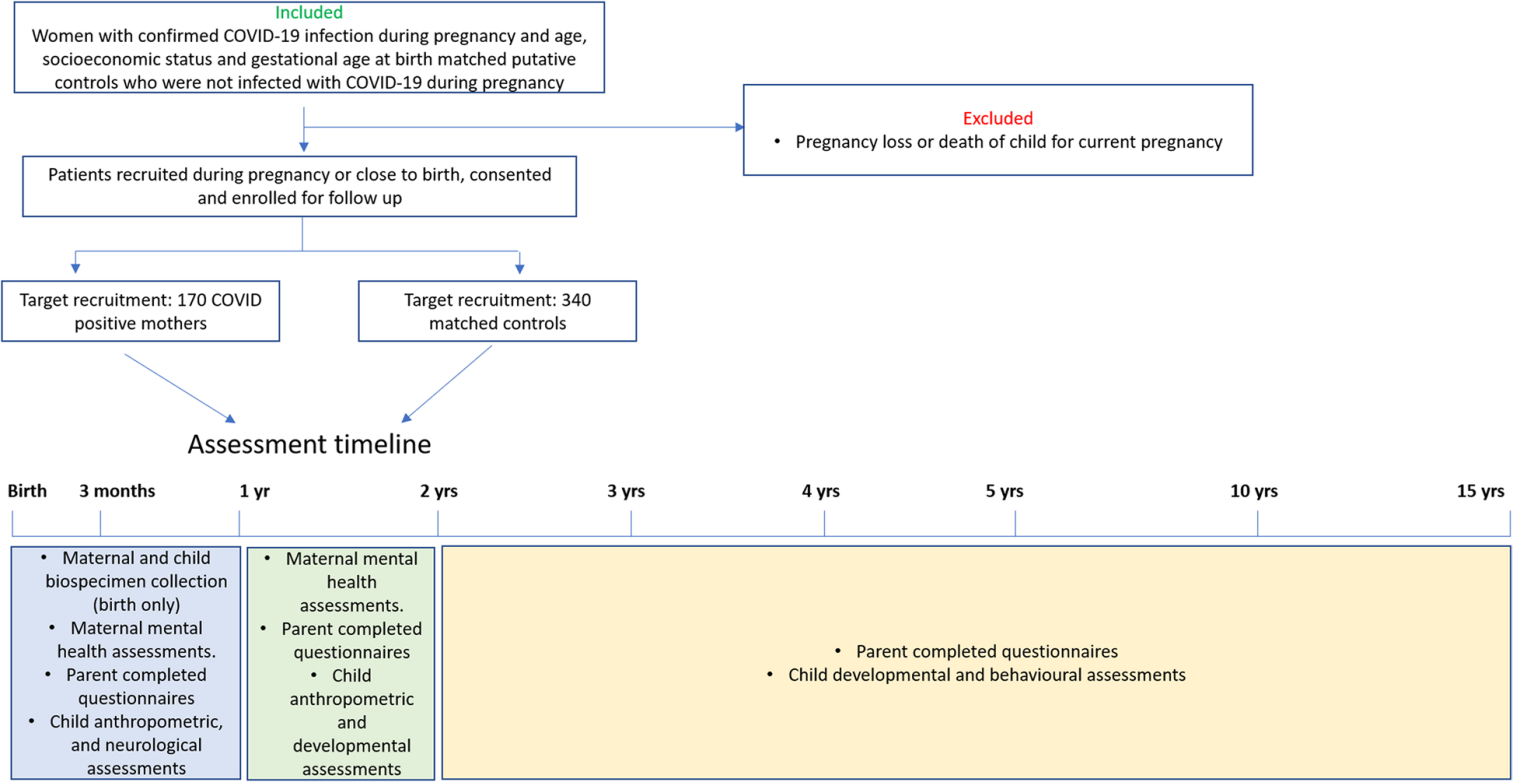
Timeline of assessments

The study is a case-controlled investigational assessment of the long-term impacts of SARS-CoV-2 in utero exposure on children from birth to 15 years old. Ethics approval has been obtained through Monash Health Human Research Ethics Committee RES-20–0000-801A (protocol #6, 17/03/2022) and the National Council of Research Ethics (CONEP, acronym in Portuguese) with protocol number 5.234.055. The study aligns with the SPIRIT guidelines. Women infected with SARS-CoV-2 during pregnancy are being recruited from Monash Health, the Royal Women’s Hospital and Sunshine Hospital (Melbourne sites), and Londrina Municipal Maternity Hospital Lucilla Ballalai and PUCPR Medical Clinic (Londrina, Brazil). A putative control group in a 2:1 ratio is also being recruited of women who gave birth in the same month of delivery, and are of similar age (within a 5-year age bracket) but who did not contract SARS-CoV-2 during their pregnancy. It is important to note here that there are currently no sufficiently specific or sensitive tests to differentiate past vaccination from past infection of COVID-19, and with vaccination rates over 95% in Australia we cannot definitively test if a mother has had a COVID-19 infection during their pregnancy. Therefore, this group is a putative control group based on whether the mother has reported infection with COVID-19 during the pregnancy or not. Demographic information is collected from the mother at the first visit. See Table 1 for the complete list of demographic data collected. Exclusion criteria are loss of pregnancy. Multiple births (twins) are included and matched to non-exposed multiple births. Assessments are planned at birth, 3 months, 1, 2, 3, 4, 5, 10 and 15 years (see Fig. 1 Timeline diagram).

**Table 1:** Maternal and child demographics

Maternal Demographics
Age (years)
Number of previous pregnancies
Educational attainment: High school or less / Diploma/TAFE / University degree or higher
Employment status
Language spoken at home
Marital status
Residential postcode
Migration status (last 5 years)
Past medical conditions
Medical conditions of 1^st^ degree relatives
Gestation of infection: 1^st^, 2^nd^ or 3^rd^ trimester
Pregnancy complications
Mode of delivery: Normal vaginal delivery / Caesarean section
History of mental illness
History of substance abuse
History of domestic violence
Vaccination status: 1, 2 or 3 doses; brand of vaccine; adverse reactions to vaccine
Dominant variant at time of infection
Medications received for infection
Child Demographics
Sex
Gestational age at birth
Neonatal Intensive Care Unit (NICU) admission
Only child or siblings
Medical conditions of siblings

### Maternal study specific data

1. For women who tested positive for COVID-19: timing of the illness (weeks of pregnancy), highest temperature recorded during illness, duration of illness and symptom severity (World Health Organisation (WHO), seven-point ordinal scale) [19], disease modifying treatments received are recorded. COVID-19 vaccination status at the first appointment is also recorded. If the participant is vaccinated, the date of each vaccination and brand of vaccine is recorded.
2. All mothers will complete the Edinburgh Postnatal Depression Scale (EPDS). The EPDS is a questionnaire designed to screen women for symptoms of emotional distress during pregnancy and the postnatal period [20]. This is a 10-question survey which takes approximately 5–10 min to complete. This test will be completed at the initial birth assessment as well as the 3 and 12-month follow up assessments and will be administered by the study coordinator.
3. All mothers will complete the Maternal Postnatal Attachment Scale (MPAS). The MPAS is 19-item self-report questionnaire to measure a mother’s subjective feelings of attachment to her infant [21]. The MPAS will be completed at birth, 3 month and 12-month time points and should take approximately 10–15 min to complete, and will be administered by the study coordinator.

### Parent-completed questionnaires about the child

All parent-completed questionnaires are administered by the clinical trials coordinator through either Q Global web-based platform for test administration (Pearson Clinical Assessment, Sydney, NSW, Australia), ACER, Melbourne, Australia or Psychological Assessments Australia, NSW, Australia. A trained Allied health professional (psychologist / neuropsychologist) will score and interpret the data.

At birth, 3 and 12 months the following questionnaires will be administered:

- 1. The Vineland adaptive behaviour scale—Third Edition (VABS-3) is an assessment of the child’s adaptive functioning [22]. It assesses 4 domains: communication, daily living, socialisation and motor skills. The VABS takes approximately 15–20 min to complete and will be administered through Q Global web-based platform for test administration (Pearson Clinical Assessment, Sydney, NSW, Australia).
- 2. Sensory Profile-2 (SP-2 questionnaire) [23]: an assessment of the child’s sensory processing patterns to understand how they may be impacting their participation in home, school and community-based activities. It takes approximately 5–20 min to complete and will again be administered through the Q global web-based platform.

At 2 and 3 years of age the VABS-3 and SP-2 will be administered as well as the Child Behaviour Checklist (CBCL) and the Repetitive Behavior Scale-Revised (RBS-R).

The CBCL: Preschool Version assesses specific kinds of behavioural, emotional and social difficulties that can be experienced by pre-school and school-aged children [24]. The questionnaire is completed by parents and takes approximately 10–20 min to complete (ACER, Melbourne, Australia).

The RBS-R is a 43 item questionnaire that assesses presence and severity of stereotyped behaviour, self-injurious behaviour, compulsive behaviour, routine behaviour, sameness behaviour, and restricted behaviours, which are associated with autism [25]. The questionnaire takes approximately 5–15 min to complete and will be administered through the Q Global web-based platform.

At 4 years of age, the VABS-3, SP2, CBCL and RBS-R will be administered as well as the Children’s Communication Checklist – Second Edition (CCC-2). The CCC-2 screens children who are likely to have communication difficulties and pragmatic language impairments [26]. The questionnaire takes approximately 5–15 min to complete and will be administered through the Q global web-based platform.

At 5 years of age, the VABS-3, SP2, CBCL, RBS-R and CCC-2 will be administered as well as the Behaviour Rating Inventory of Executive Functioning (BRIEF) (child version). The BRIEF assesses aspects of executive functioning as observed in the home environment.

[27] (Psychological Assessments Australia, NSW, Australia).

At 10 and 15 years of age, the VABS-3, SP2, CBCL, RBS-R, CCC-2 and BRIEF (child version) will be administered as well as the Connors 3^rd^ Edition-Parent assessment of Attention Deficit / Hyperactivity disorder [28]. The Connors assessment is commonly used to assess for ADHD and its common comorbidities in children aged 6 to 18 years.

### Study-specific data collected from the infant

#### Birth time point

The following information will be collected at birth (or within gestational ages 40–44 weeks):

- 1. Anthropometry: weight, length, and head circumference.
- 2. Hammersmith Neonatal Neurological Examination (HNNE). The HNNE is a 34-item examination assessing tone, motor patterns, observation of spontaneous movements, reflexes, visual and auditory attention and behaviour [29]. This assessment will be scored by a health professional trained in the administration of the HNNE who is blinded of the maternal COVID-19 status, and takes approximately 10–15 min.
- 3. General movements assessment (GMA). The GMA is used to identify normal writhing, or abnormal cramped synchronised, poor repertoire or chaotic movements [30]. The assessment is scored from a 3–5 min video of the infant while they are lying on their back in a calm but alert state. This assessment will be scored by a health professional trained in the administration of GM’s who is blinded of the maternal COVID-19 status.

#### 3 months (corrected age) time point

At 3 months of age (corrected for prematurity) anthropometry (weight, length, head circumference) will be recorded as well as the GMA and the Hammersmith Infant Neurological Examination (HINE). At 3 months of age the GMA is used to assess normal fidgety or absent or abnormal movement. The HINE is a neurological assessment for infants aged between 2 and 24 months. The assessment includes a neurological examination which is scored, developmental milestones and behaviour (which are not scored) [31]. The neurological examination consists of 26 items from 5 domains, including cranial nerve function, posture, quality and quantity of movements, muscle tone, and reflexes and reactions. The GMA and HINE at 3 months will be scored by a health professional trained to administer these assessments and who is blinded of the maternal COVID-19 status.

#### 12 months (corrected age) time point

At 12 months of age, anthropometric data are collected. In addition, the following scales are administered by a trained allied health professional:

1. The Bayley’s Scale of Infant and Toddler Development Fourth Edition (BSID IV), which is a test of development quotient [32].
2. The Ages and Stages Questionnaire (ASQ-3) as well as the ASQ: social and emotional 2. This questionnaire is a developmental screening tool for children aged between one month to 5 1/2 years [33].

#### 24 months (corrected age) time point

At 24 months of age anthropometric data will be collected and a medical examination for vision, hearing and cerebral palsy is conducted. In addition, the following scales are administered by trained health professionals (psychologist and speech pathologist) who are blinded of the maternal COVID-19 status:

- 1. Bayley’s Scale of Infant and Toddler Development Fourth Edition (BSID IV).
- 2. The Autism Diagnostic Observation Schedule-Second Edition (ADOS-2) [34]
- 3. Preschool Language Scales-Fourth Edition (PLS-4) [35], a test for communication skills.

#### 3-year time point

At 3 years of age, the BSID IV, ADOS-2 and PLS-4 will be administered (as above at the 2-year time point).

#### 4-year time point

At 4 years of age, the ADOS-2 and PLS-4 as well as the Stanford-Binet Intelligence Scale (SBIS) [36] – intelligence quotient, will be administered by trained health professionals.

#### 5-year time point

At 5 years of age, the ADOS-2 and SBIS as well as the Clinical Evaluation of Language Fundamentals- Fourth Edition (CELF-4) [37] will be administered by trained health professionals. The CELF tests for communication and language skills for children 5 years and older.

#### 10 and 15-year time point

At 10 years and at 15 years of age, the ADOS-2, SBIS and CELF-4 will be administered by trained health professionals.

### Optional biospecimen collection

#### Maternal biospecimen collection

For mothers who consent to biospecimen sample collection we will access their bio-banked samples collected during their infectious period. Blood samples and nasal mucosa will be assessed for viral load and inflammatory and cytokine marker analysis. If the mother has recovered prior to study participation biospecimens, including blood, saliva and buccal swabs, will be collected upon first visit. Blood samples will be collected by a health professional and assessed for levels of inflammatory markers [22]. Saliva will be collected to assess levels of cortisol [23]. Saliva samples are collected by the participant as soon as they wake, on the morning of their first assessment, in order to capture the waking cortisol response. Buccal swabs will be collected by a health professional and DNA will be extracted for epigenetic analysis.

#### Infant biospecimen collection

Parents may consent to provide a buccal swab sample from the infant. Biospecimens will be collected by a health professional at birth (or near the expected due date if born preterm). DNA will be extracted from buccal swabs for epigenetic analysis. At the time of birth, mothers who have a caesarean birth will also be given the option to consent to the collection of the umbilical cord blood and placental tissue. In cord blood and placental tissue, we will assess viral load (if infection was close to the time of birth), inflammatory and cytokine markers, mitochondrial function, and indices of mitochondrial structure and function. Additionally, placental morphology will be assessed using routine histopathological methods. Umbilical cord blood and stem and progenitor cell composition will be determined using flow cytometry.

## Statistical analysis and power calculations

The data collected at each assessment will be compared between SARS-CoV-2 exposed and control groups longitudinally using a separate linear mixed effects analysis for each outcome measure. Given the number and frequency of measures there are likely to be missing datapoints, thus a mixed modelling approach will avoid the need for listwise deletion of incomplete data. Sociodemographic and clinical characteristics will be compared between groups using t-tests, Mann–Whitney U tests, or Chi-square tests as appropriate. If these confounders are statistically significant between the groups they will be included as covariates within the mixed modelling analysis. Machine learning approaches will be used to determine risk profiles based on demographic and biological data. Separate analysis will be done to split the COVID-19 group into those that scored higher than a 2 for illness severity (WHO ordinal scale) and those scoring under 2 (2 COVID-19 groups and 1 control group). Another analysis will split the COVID-19 group by those infected early in pregnancy (< 20 weeks) or late (> 20 weeks).

Power analysis using G*Power for an Analysis of Variance (ANOVA) repeated measure, between factor approach with 3 groups and 7 measures (7 assessment time points) suggests a sample size of 147 is required to have 95% power to detect a medium effect size of Cohen’s f = 0.25. To allow for potential dropouts we aim to recruit 170 mother-infant dyad cases and 340 mother-infant dyad controls to detect a medium effect.

## Data management plan

Biospecimens will be stored and analysed in the laboratories at Monash Health, Monash Medical Centre, Monash University Clayton. Samples collected at Sunshine Hospital or at the Royal Women’s hospital will be stored short-term at these facilities before being transferred as a cohort to Monash Health (Behavioural neuroscience laboratory, Monash University). Samples collected will be de-identified at the time of collection and allocated a study code. This means that any information which could identify the participant, such as name, address, date of birth and hospital record number will be removed before the specimen is sent to the laboratory for analysis. We expect that all the blood, saliva and buccal swabs that we collect will be used for laboratory analysis. However, after the laboratory work has been completed, if there is any sample left over, it will either be stored at Monash Medical Centre (MMC) or discarded depending on the consent completed by the participant. Here we will give the participant the option (tick box) to either consent to immediate use, then any left over to be discarded, or to long-term storage of the samples for future unspecified use related to the study.

Maternal demographics and questionnaires will be stored in a password protected file, or in a locked cabinet held at MMC. Demographic and questionnaire data will be de-identified at the time of collection and allocated a study code. This means that any information which could identify the participant, such as name, address, date of birth and hospital record number will be removed prior to analysis. Data may only be accessed by researchers listed on the proposal.

Child developmental outcomes will be stored in a password protected file, or in a locked cabinet held at Monash Medical Centre. All assessment data will be de-identified at the time of collection and allocated a study code. This means that any information which could identify the participant, such as name, address, date of birth and hospital record number will be removed prior to analysis. Data may only be accessed by researchers listed on the proposal.

### Plans for return of results of research to participants

We will generate a short summary report in lay terms following each assessment displayed as a ‘strengths and difficulties’ framework. Scores will not be shared with the parents/caregivers as assessment scores may be misinterpreted. We will ask for parent/caregivers’ permission to share data with professionals on an ‘as requested’ basis – as required for health, disability and/or education purposes.

## Discussion

The study is currently approved at Monash Health and Londrina participant health services. As Melbourne, Australia is currently experiencing a high prevalence of COVID-19 cases, thought to be attributed to the highly contagious Omicron strain, practical and operational issues to consider include hospital restrictions that discourage face to face participant involvement. Here, telehealth options have been explored, particularly for the 12-month assessments, which do not require neurological assessments, such as the Hammersmith neurological scales that must be done in person. For the birth and 3-month assessments, extra precautions have been planned, including personal protective equipment and social distancing compliance.

Another operational issue to consider is that with the high vaccination rates now in Victoria (~ 95% people aged 15 and over double vaccinated) and Brazil (~ 93% of the population have received 2 doses as at 09/03/2022), there is likely to be variation in that data, with some women having received 1, 2 or 3 doses and some being unvaccinated. This has been included in the study design as a question in the demographics; ‘Are you vaccinated? If so when? and How many doses?’. However, depending on the numbers this will need to be considered as a variable when analysing the data. We would anticipate that women who have been vaccinated will have a less severe course of illness, which will be reported through the WHO 7-point ordinal scale. These data will allow us to assess this anticipated hypothesis.

Our study design is such that biospecimen samples are collected at birth (or as close to birth as possible), then we will map our biomarker findings onto the neurodevelopmental trajectory of the child. For some women, the collection of biospecimens will be only shortly after they have been infected with COVID-19, while for others, they may have been infected early in their pregnancy. This variation in the time since infection is a limitation of the study. However, we have also linked this project to a COVID-19 biobank established through Monash Health, which collects mucosal swabs and serum samples at the time of infection. While not all participants will consent to both studies, these data will provide us with a unique opportunity to map biomarkers during infection to the neurodevelopmental trajectory of the child.

Overall, this established protocol will allow longitudinal, prospective analysis of the neurodevelopment of children exposed in utero to SARS-CoV-2 to determine the risk that COVID-19 infection during pregnancy poses to the infant. A secondary set of outcomes will be the biological findings from our biospecimen collections and consequent mapping of biological changes on the child’s neurodevelopmental trajectory. These data may provide valuable new knowledge on biomarkers or risk pathways of neurodevelopmental disturbances. The scales used in this study have been validated across multiple cultures, ensuring global uptake feasibility. With collaborations established in Londrina, Brazil, we call for international uptake of this protocol to inform health care professionals globally of the risk of COVID-19 infection during pregnancy to the neurodevelopment of the infant.

## Data Availability

The datasets generated during and/or analysed during the current study are not publicly available yet due to the majority of the data not collected yet but are available from the corresponding author on reasonable request.

## Abbreviations

SARS-CoV2: Severe acute respiratory syndrome coronavirus 2
IgM: Immunoglobulin M
IgG: Immunoglobulin G
COVID-19: Coronavirus disease of 2019
EPDS: Edinburgh postnatal depression scale
MPAS: Maternal postnatal attachment scale
VABS-3: Vineland adaptive behavior scale – 3^rd^ edition
SP-2: Sensory profile-2
CBCL: Child behavior checklist
RBS-R: Repetitive Behavior Scale-Revised
CCC-2: Children’s communication checklist – 2^nd^ edition
BRIEF: Behaviour Rating Inventory of Executive Functioning
HNNE: Hammersmith neonatal neurological examination
HINE: Hammersmith infant neurological examination
GMA: General movement assessment
BSID IV: Bayley’s Scale of Infant and Toddler Development Fourth Edition
ASQ-3: Ages and Stages Questionnaire 3^rd^ edition
ADOS-2: Autism Diagnostic Observation Schedule-Second Edition
PLS-4: Preschool Language Scales-Fourth Edition
SBIS: Stanford-Binet Intelligence Scale
CELF-4: Clinical Evaluation of Language Fundamentals- Fourth Edition
WHO: World health organisation
ANOVA: Analysis of variance
